# *In Vitro* Antimicrobial, Antioxidant, Cytotoxicity and GC-MS Analysis of *Mazus goodenifolius*

**DOI:** 10.3390/molecules171214275

**Published:** 2012-12-03

**Authors:** Muhammad Riaz, Nasir Rasool, Iftikhar Hussain Bukhari, Muhammad Shahid, Muhammad Zubair, Komal Rizwan, Umer Rashid

**Affiliations:** 1 Department of Chemistry, Government College University Faisalabad-38000, Pakistan; E-Mails: riaz_453@yahoo.com (M.R.); pdiftikhar@yahoo.com (I.H.B.); zubairmkn@yahoo.com (M.Z.); komal.rizwan45@yahoo.com (K.R.); 2 Department of Chemistry and Biochemistry, University of Agriculture, Faisalabad-38040, Pakistan; E-Mail: mshahiduaf@yahoo.com; 3 Institute of Advanced Technology, Universiti Putra Malaysia, Serdang 43400, Selangor, Malaysia

**Keywords:** *Mazus goodenifolius*, antimicrobial, antioxidant, cytotoxicity, GC-MS

## Abstract

The antimicrobial, antioxidant and cytotoxic properties of *Mazus goodenifolius* (Hornem.) Pennell essential oil, methanol extract and some solvent-extracted subfractions of the latter were appraised. A qualitative, quantitative analysis of the classes of phytochemicals in the various fractions and GC-MS analysis of the essential oil was carried out. The activity of the plant extract and various subfractions against selected bacterial (*Pasturella multocida*, *Escherichia coli*, *Bacillus subtilis* and *Staphylococcus aureus*) and fungal strains (*Aspergillus niger*, *Aspergillus flavus*, *Alternaria alternata* and *Rhizopus solani*) was evaluated. The antioxidant activity was assayed using the DPPH radical scavenging and % inhibition of linoleic acid peroxidation tests. In the DPPH radical scavenging test the IC_50_ values ranged from 7.21 to 91.79 µg/mL, and in the latter the range of % peroxidation inhibition was 35.42–93.48%. Protective effects of the absolute methanol extract, which had the highest content of phenolics and flavonoids, against H_2_O_2_ induced oxidative damage in plasmid pBR322 DNA was also evaluated, and it was found to offer some protection at the highest tested dose (1,000 µg/mL). Finally the cytotoxicity of the plant extract, fractions and essential oil was analyzed by examining haemolytic activity against human blood erythrocytes (RBCs), whereby the % lysis of RBCs was found to be in the range of 1.65 to 4.01%.

## 1. Introduction

Foods rich in natural antioxidants such as polyphenols, flavonoids are related to reduced risk of incidence of cardiovascular and other chronic diseases and certain types of cancer, which has led to a revival of interest in plant-based foods [1]. Conversely, food-borne diseases are major dilemma in the third world and developing countries and even in developed nations [2], and the consumption of foods contaminated with microorganisms represents a serious health risk to humans. Most of the antibiotics existing today have a natural origin. Plants produce a variety of compounds to defend themselves from microbial attacks [3], and natural products and related drugs were used to treat 87% of all categorized human diseases, including bacterial infections, cancer and immunological disorders [4]. 

About eighty percent of the population in developing countries relies on traditional plant-based medicines for their primary health care needs [5]. However, the majority of plants have not yet undergone comprehensive chemical, pharmacological and toxicological studies to investigate their bioactive compounds [6]. We have now studied the plant *Mazus goodenifolius* (Hornem.) Pennell (family Scrophulariaceae) by GC-MS analysis and biological studies. According to our knowledge, to date no literature has reported the antioxidant, antimicrobial and cytotoxic properties of *Mazus goodenifolius*, although as per previous reports plants related to this genus were used as an aperients, emmenagogue, febrifuge and tonic [7]. The juice of the plant *Mazus pumilus *is used in the treatment of typhoid fever [8]. The leaves of some *Mazus* species plants are also edible. 

## 2. Results and Discussions

### 2.1. Phytochemical Analysis

In the present study, efforts were made to qualitatively estimate the various medicinally active constituents such as flavonoids, saponins, tannins, steroids, alkaloids and terpenoids present in *M. goodenifolius *extract and its various fractions. These constituents were present in all the extracts and fractions, with the exception of alkaloids, tannins and saponins which were absent in the *n-*hexane fraction, whereas saponins and steroids were absent in the *n-*butanol fraction. Quantitative estimation of phytochemicals was also carried out on dried whole plant. The findings showed that the highest percentage of components corresponded to tannins (9.12 ± 0.07%) and the lowest yield was of saponins (0.12 ± 0.01%). The percentages of other constituents were 1.44 ± 0.03 for alkaloids, 1.27 ± 0.02 for flavonoids, 0.15 ± 0.01 for terpenoids and 0.33 ± 0.02 for steroids, respectively. 

### 2.2. GC-MS Analysis of Essential Oil

The % yield of *M. goodenifolius *essential oil was found to be 0.32%. The chemical compounds identified by GC-MS analysis of the essential oil are presented in Table 1. The major compounds determined in the essential oil were: thymol (15.16%), carveol (10.06%), linalool (9.96%), *α*-copaene (9.61%), germacrene D (9.37%), 1,8-cineole (8.41%), *β*-pinene (6.43%) and γ-terpinene (5.46%), respectively. Some other compounds found in different concentrations were: *β*-ocimene (3.82%), carvacrol (2.73%), verbenone (2.67%), α-fenchone (2.62%), limonene (2.13%), α-pinene (1.79%) and *p*-cymene (1.53%). From the activity results it was observed that the antioxidant and antimicrobial activity of the *M. goodenifolius *essential oil was greater than that of the methanol extract and fractions. Some studies have also shown that plant essential oils can have greater antimicrobial activity due to the synergistic or additive effects of their components [9]. 

### 2.3. Antimicrobial Activity

The antimicrobial activity of the *M. goodenifolius *extract, fractions and essential oil was tested against selected microorganisms. The samples some exhibited antimicrobial activity against most of the bacterial and fungal strains tested. The inhibition zone (IZ) was measured by the disc diffusion method at 10 mg/mL (Table 2) and 20 mg/mL concentrations (Table 3), followed by measurement of minimum inhibitory concentrations (MICs, Table 4). 

It was observed that some of the strains were resistant at 10 mg/mL, when the concentration was increased to 20 mg/mL, inhibition zones also observed. Overall the results indicated that the the *M. goodenifolius* essential oil and the absolute methanol extract showed comparatively better inhibitory activity than the other analyzed fractions. The *n-*hexane fraction showed less/poor/no activity against the various tested bacterial and fungal strains. The minimum inhibtory concentration (MIC) also confirmed that the antimicrobial actibity of the essential oil was better than that of the extract and fractions. The secondary metabolites identified during the phytochemical assay in the plant are steroids, tannins, saponins, flavonoids, alkaloids and terpenoids. These compounds have been variously reported to showed antimicrobial activity [10]. The better antimicrobial activity of the essential oil was attributed to the presence of some of its major components thymol, carveol, linalool, germacrene D, 1,8-cineole, β-pinene, γ-terpinene, β-ocimene, carvacrol, limonene, α-pinene and *p*-cymene. Essential oils containing these major compounds have been reported to show antimicrobial properties [11,12,13]. The synergistic or antagonistic activity between some components may affect the observed antimicrobial activity of the essential oil [14], which exerts its toxic effects against microorganisms through the disruption of bacterial and fungal membrane integrity [15].

### 2.4. Antioxidant Activity

**Figure 1:**
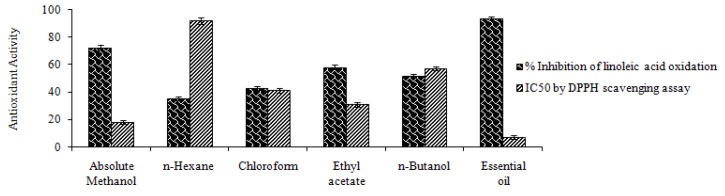
Antioxidant activity of *M. goodenifolius* extract, fractions and essential oil by % inhibition of linoleic acid and IC_50_ by DPPH scavenging assay.

The antioxidant activity of plant extract, fractions and essential oil was determined. The plant showed % inhibition of linoleic acid peroxidation values ranging from 35.44 to 93.48. The % inhibition of peroxidation for the essential oil, absolute methanol extract, ethyl acetate, *n*-butanol, chloroform, *n*-hexane fractions was 93.48 ± 0.82, 72.35 ± 0.68, 58.21 ± 0.14, 51.62 ± 0.52, 42.81 ± 0.34 and 35.44 ± 0.29%, respectively (Figure 1). The highest % inhibition in linoleic acid peroxidation was thus observed in the essential oil. 

The DPPH radical, which has a deep violet color, reacts with hydrogen donor species such as phenolics, flavonoids and upon receiving a proton loses its color and becomes yellow. The IC_50_ values for essential oil, absolute methanol, ethyl acetate, *n*-butanol, chloroform and *n*-hexane extract were: 7.21 ± 0.06, 9.96 ± 0.08, 18.23 ± 0.12, 31.01 ± 0.29, 41.18 ± 0.32, 57.16 ± 0.42 and 91.79 ± 0.89 µg/mL, respectively. The smaller value of IC_50_ represents a better antioxidant activity.

Derwich and coworkers previously reported that 1,8-cineole, germacrene, limonene, pulegone *β*-pinene and *α*-pinene are all good antioxidants [16]. Thymol also behaves as an antioxidant [17]. As per earlier reports it was observed that the presence of chemical constituents such as linalool, *β*-pinene and α-pinene in essential oil also resulted in antioxidant and antimicrobial properties [11,12].

**Figure 2:**
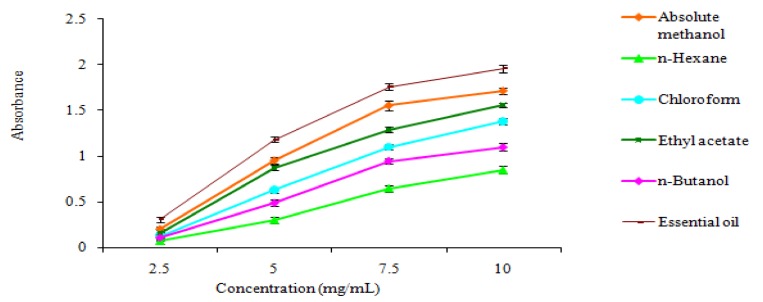
Reducing power of extract, fractions and essential oil of *M. goodenifolius*.

The reducing power of *M. goodenifolius *plant extract, fractions and essential oil is shown in Figure 2. The reducing power of the phytoconstituents is associated with their antioxidant potential [18]. The reducing power of the extracts increased in a concentration-dependent manner [19]. Therefore reducing power evaluation might be taken as important parameter for the assessment of antioxidant activity.

### 2.5. Antioxidant Activity by DNA Protection Assay

**Figure 3:**
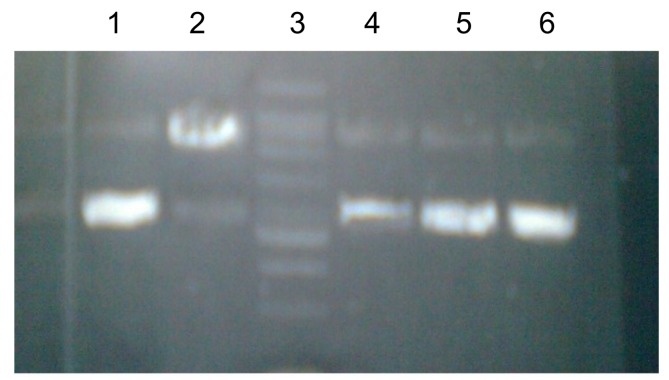
DNA protection effect by absolute methanol extract with H_2_O_2_ induced oxidative damage on pBR322 DNA.

The antioxidant activity of different concentrations of *M. goodenifolius *absolute methanol extract in the protection of plasmid pBR322 DNA from H_2_O_2_ induced damage is shown in Figure 3. From the figure it was clear that in the first lane the plasmid pBR322 DNA present without any treatment might be in super coiled form. When comparing the results of the second lane with the other lanes, which contain pBR322 DNA that was exposed to H_2_O_2_, that caused damage in plasmid pBR322 DNA, in the second lane the damage to the DNA strand, which occured due to conversion of the super coiled form of pBR322 DNA into an open linear form, leaving behind the untreated DNA (first lane) can be seen. In the fourth to sixth lane 10 to 1,000 µg/mL of the plant absolute methanol extract was added in pBR322 DNA to observe their protective effects. The results in Figure 3, when compared with each other, show the protective effects of the absolute methanol extract at a concentration of 1,000 µg/mL (sixth lane) which displays a band almost equal to the pure pBR322DNA (first lane). The absolute methanol extract at a concentration of 10 µg/mL (fourth lane) showed less protective effect on DNA and the band in this lane was similar to the damaged DNA (second lane treated with H_2_O_2_).

The plant extract at 1,000 µg/mL protected the DNA, perhaps by scavenging the oxidation products that damage the DNA and this did not allowed the H_2_O_2_ to open the coiled DNA so it remained in protected form. The protective effect by absolute methanol extract could be due to the higher concentration of phenolics, flavonoids and the antioxidant activity that scavenges the free radicals and oxidation products. 

### 2.6. Cytotoxicity Studies by Haemolytic Activity

The cytotoxicity was studied by examining haemolytic activity against human red blood cells (RBCs) using Triton X-100 as positive control. The percentage lysis evaluated by comparing the absorbance of sample and the Triton X-100. The positive control showed about 100% lysis, whereas the phosphate buffer saline (PBS) showed no lysis of RBCs.

**Figure 4:**
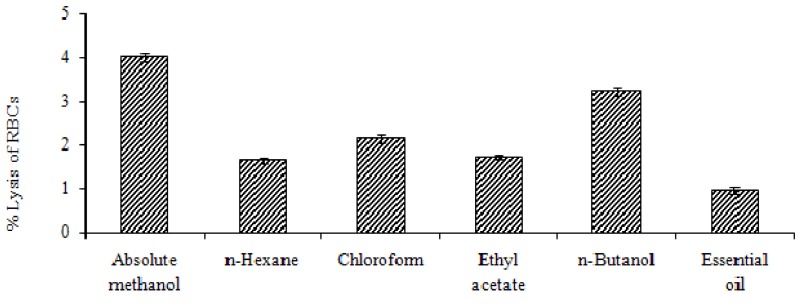
Cytotoxicity assay by heamolytic activity by extract, fractions and essential oil.

When the effects of extract, fractions and essential oil of the plant were compared with the controls, different % lysis of RBCs caused by plant samples was observed, such as absolute methanol extract (4.01 ± 0.03), *n-*butanol (3.24 ± 0.03), chloroform (2.15 ± 0.02), ethyl acetate (1.73 ± 0.02) and *n-*hexane (1.65 ± 0.01) fractions, essential oil (0.96 ± 0.01), respectively (Figure 4). The mechanical stability of the membrane of red blood cells (RBCs) is a good indicator to evaluate *in vitro *the effects of various compounds when screening for cytotoxicity [20,21]. Treating cells with a cytotoxic compound can cause different problems to human beings. The cells may undergo a loss of membrane integrity and die rapidly as a result of cell lysis [22]. 

## 3. Experimental

### 3.1. General

*M. goodenifolius *(Hornem) (family Scrophulariaceae) whole plant was collected from the Botanical Garden, University of Agriculture, Faisalabad (Pakistan) in March, 2011. The plant was further identified and authenticated by the taxonomist Dr. Mansoor Hameed, Department of Botany, University of Agriculture, Faisalabad. The voucher specimen *(4182) was submitted to the* herbarium/collection at Department of Botany, University of Agriculture, Faisalabad. After collection the plant material (5 Kg) was washed, shade dried and ground. The whole plant was extracted thrice with absolute methanol (3 × 7 L) by dipping for seven days each time, then the extracts were combined and concentrated to dryness under reduced pressure using a rotary evaporator (Heidolph, Schwabach, Germany). The absolute methanol extract was further fractioned with solvents of increasing polarity such as *n-*hexane, chloroform, ethyl acetate and *n-*butanol, using a solvent extraction process according to reported methods [23,24]. The corresponding % yield of extract, fractions were: absolute methanol extract 17.21%, ethyl acetate 5.56%, *n*-butanol 4.38%, chloroform 4.25% and *n*-hexane 0.97%. After fractionation, samples were concentrated to dryness and stored in a refrigerator at −4 °C, until used for analysis. The reference chemicals such as homologous series of C_9_–C_24_ alkanes used for the identification were purchased from Sigma Chemical Co. (St. Louis, MO, USA). All other chemicals used were of analytical grade and purchased from Merck (Darmstadt, Germany).

### 3.2. Phytochemical Analysis

Phytochemical analysis was carried out by using the standard procedures to identify the constituents qualitatively in plant extracts, fractions and quantitatively in dried whole plant as described by Edeoga *et al.* [25].

### 3.3. Isolation of Essential Oil

The dried and ground whole plant (500 g) was hydro-distilled for four hours using a Clevenger-type apparatus as described earlier [11,12]. The percentage yield of essential oil was found to be 0.32%. The essential oil was collected and dried over anhydrous sodium sulfate, filtered and stored at 4 °C until analyzed.

### 3.4. GC-MS Analysis of Essential Oil

The sample was analyzed using a GC 6850 network GC system equipped with a 7683B series auto injector and 5973 inert mass selective detector (Agilent Technologies, Willmington, DE, USA). Compounds were separated on an HP-5 MS capillary column with a 5% phenyl polysiloxane stationary phase (30.0 m × 0.25 mm, film thickness 0.25 μm). Oven temperature was programmed in a three step gradient: initial temp set at 45 °C (held for 5 min), ramped till 150 °C at 10 °C/min, followed by a 5 °C/min rise till 280 °C and finally at 15 °C/min to 325 °C where it was held for 5 min. Helium gas flow rate was 1.1 mL/min (pressure 60 KPa and linear velocity 38.2 cm/sec). Ions/fragments were monitored in scanning mode through 40–550 *m/z*.

### 3.5. Identification of Compounds

The identification of the components was based on comparison of their retention index (RI), relative to a standard alkane series (C_9_–C_24_). The compounds were further indentified and authenticated using their MS data by comparison with those of the NIST 05 Mass Spectral Library and published mass spectra [26]. The quantitative data were obtained electronically from the FID area percentage without the use of any correction factors.

### 3.6. Antimicrobial Activity

In order to evaluate the antimicrobial activity of plant extract, fractions and essential oil at different concentrations of 10 and 20 mg/mL against selected bacterial strains such as *Pasturella multocida, Escherichia coli, Bacillus subtilis *and* Staphylococcus aureus *and the fungal strains *Aspergillus niger*, *Aspergillus flavus*, *Alternaria alternata *and *Rhizopus solani *the disc diffusion method as described by the CLSI was used [27]. Minimum inhibitory concentrations (MIC) were calculated by a modification of the reported method of Sarker *et al.* [3]. For the evaluation of minimum inhibitory concentrations (MIC), different concentrations of plant extract, fractions and essential oil were prepared by serial dilution. The range of dilution was determined by keeping in mind the antimicrobial activity determined in the inhibition zone assay. For the samples showing better activity in the first assay the serial dilution for MIC determination was carried out at less concentration and for the samples showing less activity the higher concentration was used for serial dilution. Ciprofloxacin and fungone at 1,000 µg/mL were used as reference standards for the bacterial and fungal strains, respectively.

### 3.7. Antioxidant Activity

#### 3.7.1. DPPH free Radical Scavenging Assay

For the determination of IC_50_ values by the DPPH free radical scavenging assay method described by Iqbal *et al. *[28] with some modification was used. The IC_50_ values were calculated from the plot of the regression equation against percentage scavenging and concentrations of samples used. Three replicates were recorded for each sample. The percentage scavenging by DPPH was calculated from the following equation:

Scavenging (%) = [(A_blank_ − A_sample_) / A_blank_] × 100

where A is the absorbance.

#### 3.7.2. Percentage Inhibition of Linoleic Acid Oxidation

The antioxidant activity of extract, fractions and essential oil was also calculated in terms of percentage inhibition of oxidation in the linoleic acid system using a method with some modification described by Iqbal *et al.* [28]. A sample that contained no antioxidant component was used as a blank. Percentage inhibition of linoleic acid oxidation was determined by the following equation:

Inhibition (%) = [100 − (A increase of sample at 360 h/A increase of control at 360 h) × 100]

where A is the absorbance.

#### 3.7.3. Determination of Reducing Power

The reducing power of the extract, fractions and essential oil was determined according to the procedure described by Yen *et al.* [29]. Plant extract, fractions and essential oil samples containing 2.5–10.0 mg/mL of dry matter was mixed with sodium phosphate buffer (5.0 mL, 0.2 M, pH 6.6) and potassium ferricyanide (5.0 mL, 1.0%); the mixture was incubated at 50 °C for 20 min, then 10% trichloroacetic acid (5 mL) was added, and the mixture was centrifuged at 980 *× g* for 10 min at 5 °C in a refrigerated centrifuge. The upper layer of the solution (5.0 mL) was diluted with distilled water (5.0 mL), 0.1% ferric chloride (1 mL) was added and the absorbance at 700 nm noted. 

#### 3.7.4. Antioxidant Activity by DNA Protection Assay

To evaluate the antioxidant activity of the the absolute methanol extract by the DNA protection assay the method described by Kalpana *et al.* was used [30]. pBR 322 DNA (0.5 µg/µL) was diluted up to two-fold (0.5 µg/3 µL) using 50 mM pH 7.4 sodium phosphate buffer. The diluted pBR 322 DNA (3 µL) was treated with test sample (5 µL). After this 30% H2O2 (4 µL) was added in the presence and absence of different concentrations of *M. goodenifolius *extracts and its fractions. The volume was made up to 15 µL with sodium phosphate buffer (pH 7.4). Samples of absolute methanol extract of different concentrations (1,000, 100 and 10 mg/L) were used. The relative difference in migration between the native and oxidized DNA was then examined on 1% agarose by horizontal DNA gel electrophoresis using a Bio-Rad wide mini system (Techview, Singapore). The gels were documented by a Syngene model Gene Genius unit (Syngene, Cambridge, UK).

### 3.8. Cytotoxicity Studies

The cytotoxicity was determined by testing the haemolytic activity of the plant extract, fractions and essential oil using the method with some modification described by Powell *et al.* [20]. Plant extracts at a concentration of 1 mg/mL in 10% DMSO were prepared. Three mL of freshly obtained human blood was placed in heparinized tubes to avoid coagulation, gently mixed and poured into a sterile 15 mL Falcon tube and centrifuged for 5 min at 850 *× g*. The supernatant was poured off and RBCs were washed three times with chilled (4 °C) sterile isotonic phosphate buffer saline (PBS) solution (5 mL), adjusted to pH 7.4. The washed RBCs were suspended in chilled PBS (20 mL). Erythrocytes were counted on a heamacytometer. The RBCs count was maintained to 7.068 × 10^8^ cell per mL for each assay. The plant extract, fractions and essential oil (20 µL) were taken in 2 mL Eppendorf tubes, then diluted blood cell suspension (180 µL) was added. The samples were incubated for 35 min at 37 °C. After incubation and agitation for 10 min, the tubes were placed on ice for 5 min and centrifuged for 5 min at 1,310 *× g*. After centrifugation supernatant (100 µL) was taken from the tubes, and diluted with chilled PBS (900 µL). All Eppendorfs were maintained on ice after dilution. After this mixture from each Eppendorf (200 µL) was added into 96 well plates. For each assay, 0.1% Triton X-100 was taken as a positive control and phosphate buffer saline (PBS) as a negative control. The absorbance was noted at 576 nm with a BioTek, µ Cuant^TM^ instrument (BioTek, Winooski, VT, USA).

### 3.9. Statistical Analysis

All the experiments were conducted in triplicate unless stated otherwise stated and statistical analysis of the data was performed by analysis of variance, using the STATISTICA 5.5 (Stat Soft Inc, Tulsa, OK, USA) software. A probability value of difference *p* ≤ 0.05 was considered to denote a statistically significance. All data were presented as mean values ± standard deviation (SD).

## 4. Conclusions

From the results it was concluded that the essential oil of *M. goodenifolius *has comparatively greater antioxidant and antimicrobial activity than the absolute methanol extract and fractions. The GC-MS profiling showed that the essential oil has some phytoconstituents which may be responsible for the antioxidant and antimicrobial activity. DPPH scavenging and % inhibition linoleic acid peroxidation assays were performed. The protective effect of the absolute methanol extract against H_2_O_2_ induced oxidative damage in plasmid pBR322 DNA was also evaluated and it was found that it protected the DNA, probably due to its antioxidant activity. The cytotoxicity of plant extract, fractions and essential oil were assayed using *in vitro* haemolytic activity against human blood erythrocytes (RBCs) and it was observed that the plant shows minor cytotoxicity as compared to the positive control.

## Figures and Tables

Lane 1 = Plasmid pBR322 DNA without treatment (super coiled); Lane 2 = Plasmid pBR322 DNA treated with H_2_O_2_ (open circular or damaged); Lane 3 = 1 kbp DNA ladder; Lane 4 = Plasmid pBR322 DNA treated with 10 µg/mL absolute methanol extract + H_2_O_2_; Lane 5 = Plasmid pBR322 DNA treated with 100 µg/mL absolute methanol extract + H_2_O_2_; Lane 6 = Plasmid pBR322 DNA treated with 1,000 µg/mL absolute methanol extract + H_2_O_2_.

**Table 1:** Compounds identified in the essential oil of *M. goodenifolius* and their percentage area.

Compounds	Retention Index (RI)	Area percentage (%)
α-Thujene	933	0.33
α-Pinene	940	1.79
α-Fenchone	951	2.62
Camphene	953	0.48
*β*-Pinene	970	6.43
*p*-Cymene	1025	1.53
Limonene	1028	2.13
1,8-Cineole	1030	8.41
*β-*Phellandrene	1033	2.02
*β*-ocimene	1049	3.82
γ-Terpinene	1058	5.46
Linalool	1098	9.61
Carveol	1138	10.06
Verbenone	1204	2.67
Thymol	1291	15.16
2-Undecanone	1293	1.58
Carvacrol	1297	2.73
Geranic acid	1365	1.97
α-Copaene	1411	0.44
*β*-Caryophyllene	1417	9.96
α-Guaiene	1439	0.7
α-Humulene	1451	0.73
Germacrene D	1483	9.37

Mode of identification = RI and comparison of mass spectra.

**Table 2:** Antimicrobial activity in terms of inhibition zone (mm) of *M. goodenifolius* plant extract, fractions at 10 mg/mL against selected bacterial and fungal strains.

	Extract, fractions and essential oil						
	Abs. MeOH	*n-*Butanol	Chloroform	Ethyl acetate	*n-*Hexane	Essential oil	Standard ^†^
**Bacterial strains**							
***E. coli***	12.4 ± 0.11 ^c^	N.D. *^g^	9.4 ± 0.08 ^e^	10.2 ± 0.09 ^d^	8.2 ± 0.07 ^f^	16.2 ± 0.15 ^b^	28.3 ± 0.15 ^a^
***P. multocida***	14.3 ± 0.12 ^c^	8.2 ± 0.07 ^f^	10.2 ± 0.09 ^e^	13.2 ± 0.11 ^d^	N.D. ^g^	19.5 ± 0.17 ^b^	27.2 ± 0.21 ^a^
***S. aureus***	16.7 ± 0.12 ^c^	N.D. ^e^	N.D. ^e^	12.4 ± 0.09 ^d^	N.D. ^e^	22.9 ± 0.17 ^b^	30.1 ± 0.16 ^a^
***B. subtilis***	13.2 ± 0.12 ^c^	9.2 ± 0.07 ^e^	N.D. ^g^	12.3 ± 0.09 ^d^	8.2 ± 0.06 ^f^	17.2 ± 0.14 ^b^	29.2 ± 0.15 ^a^
**Fungal strains**							
***A. flavus***	10.2 ± 0.09 ^c^	7.8 ± 0.06 ^f^	8.7 ± 0.05 ^e^	9.5 ± 0.11 ^d^	N.D. ^g^	14.2 ± 0.12 ^b^	22.5 ± 0.18 ^a^
***A. alternata***	12.3 ± 0.11 ^c^	8.8 ± 0.07 ^f^	N.D. ^g^	11.3 ± 0.12 ^d^	9.4 ± 0.08 ^e^	16.2 ± 0.14 ^b^	24.6 ± 0.23 ^a^
***R. solani***	15.9 ± 0.12 ^c^	N.D. ^g^	11.1 ± 0.13 ^e^	12.2 ± 0.09 ^d^	8.4 ± 0.07 ^f^	18.5 ± 0.13 ^b^	29.7 ± 0.21 ^a^
***A. niger***	14.3 ± 0.13 ^c^	8.6 ± 0.07 ^e^	N.D. ^g^	11.4 ± 0.06 ^d^	7.8 ± 0.05 ^f^	17.9 ± 0.15 ^b^	28.1 ± 0.26 ^a^

* N.D. = Not detected. The values were the average of triplicate samples (n = 3) ± S.D. (*p* ≤ 0.05). The superscript letters represent the significant differences as analyzed by ANOVA; ^†^ The standard was used at a concentration of 1 mg/mL.

**Table 3:** Antimicrobial activity in terms of inhibition zone (mm) of *M. goodenifolius* plant extract and fractions at 20 mg/mL against selected bacterial and fungal strains.

	Extract, fractions and essential oil						
	Abs. MeOH	*n-*Butanol	Chloroform	Ethyl acetate	*n-*Hexane	Essential oil	Standard ^†^
**Bacterial strains**							
***E. coli***	25.1 ± 0.21 ^c^	16.3 ± 0.21 ^f^	19.2 ± 0.18 ^e^	20.1 ± 0.15 ^d^	15.2 ± 0.13 ^g^	30.2 ± 0.27 ^a^	28.3 ± 0.15 ^b^
***P. multocida***	28.1 ± 0.21 ^c^	15.2 ± 0.17 ^f^	19.3 ± 0.14 ^e^	26.2 ± 0.19 ^d^	12.2 ± 0.10 ^g^	32.2 ± 0.26 ^a^	27.2 ± 0.21 ^b^
***S. aureus***	31.7 ± 0.12 ^b^	10.6 ± 0.08 ^d^	14.1 ± 0.12 ^d^	23.1 ± 0.29 ^c^	N.D. ^d^	35.2 ± 0.19 ^a^	30.1 ± 0.16 ^c^
***B. subtilis***	25.4 ± 0.21 ^c^	17.2 ± 0.16 ^e^	15.7 ± 0.08 ^f^	23.3 ± 0.17 ^d^	11.2 ± 0.14 ^g^	32.2 ± 0.26 ^a^	29.2 ± 0.15 ^b^
**Fungal strains**							
***A. flavus***	19.0 ± 0.13 ^c^	14.8 ± 0.11 ^f^	17.2 ± 0.13 ^e^	18.6 ± 0.14 ^d^	N.D. ^g^	29.2 ± 0.27 ^a^	22.5 ± 0.18 ^b^
***A. alternata***	23.1 ± 0.21 ^c^	15.6 ± 0.14 ^e^	12.7 ± 0.07 ^f^	20.3 ± 0.22 ^d^	11.4 ± 0.17 ^g^	30.4 ± 0.24 ^a^	24.6 ± 0.23 ^b^
***R. solani***	30.1 ± 0.23 ^b^	N.D. ^g^	19.1 ± 0.21 ^e^	22.3 ± 0.17 ^d^	15.2 ± 0.16 ^f^	32.4 ± 0.31 ^a^	29.7 ± 0.21 ^c^
***A. niger***	28.6 ± 0.21 ^b^	14.6 ± 0.14 ^e^	N.D. ^g^	20.2 ± 0.21 ^d^	13.2 ± 0.12 ^f^	31.8 ± 0.30 ^a^	28.1 ± 0.26 ^c^

* N.D. = Not detected. The values are the average of triplicate samples (n = 3) ± S.D. (*p* ≤ 0.05). The superscript letters represent the significant differences as analyzed by ANOVA; ^†^ The standard was used at a concentration of 1 mg/mL.

**Table 4:** Antimicrobial activity in terms of minimum inhibitory concentration (MIC) in µg/mL by *M. goodenifolius* plant against selected bacterial and fungal strains.

	Extract, fractions and essential oil						
	Abs. MeOH	*n-*Butanol	Chloroform	Ethyl acetate	*n-*Hexane	Essential oil	Standard ^†^
**Bacterial strains**							
***E. coli***	1175.0 ± 9.2 ^e^	3000.0 ± 15.4 ^a^	2000.0 ± 14.1 ^c^	1250.0 ± 8.5 ^d^	2500.0 ± 16.7 ^b^	187.0 ± 1.49 ^f^	75.0 ± 0.29 ^g^
***P. multocida***	875.0 ± 6.7 ^e^	1750.0 ± 10.7 ^b^	1500.0 ± 2.8 ^c^	1150.0 ± 11.4 ^d^	2000.0 ± 11.9 ^a^	156.0 ± 1.4 ^f^	125.0 ± 1.2 ^g^
***S. aureus***	750.0 ± 6.9 ^d^	2500.0 ± 9.2 ^a^	1500.0 ± 7.2 ^b^	1000.0 ± 8.2 ^c^	N.D. ^g^	62.0 ± 0.53 ^e^	25 ± 0.13 ^f^
***B. subtilis***	1000.0 ± 8.4 ^d^	1500.0 ± 10.8 ^c^	2000.0 ± 12.1 ^a^	1250.0 ± 11.4 ^d^	1750.0 ± 18.2 ^b^	78.0 ± 0.62 ^f^	62.5 ± 0.59 ^g^
**Fungal strains**							
***A. flavus***	1750.0 ± 17.2 ^d^	3000.0 ± 15.2 ^a^	2500.0 ± 16.4 ^b^	2000.0 ± 16.8 ^c^	N.D. ^g^	125.0 ± 1.14 ^e^	100.0 ± 1.04 ^f^
***A. alternata***	1250.0 ± 13.7 ^e^	2500.0 ± 12.4 ^b^	3000.0 ± 16.6 ^a^	1500.0 ± 19.2 ^d^	1750.0 ± 21.4 ^c^	93.5 ± 0.84 ^f^	62.5 ± 0.54 ^g^
***R. solani***	1150.0 ± 10.4 ^d^	N.D. ^g^	1500.0 ± 11.6 ^b^	1250.0 ± 13.7 ^c^	1750.0 ± 14.3 ^a^	25.0 ± 0.22 ^e^	15.5 ± 0.12 ^f^
***A. niger***	1750.0 ± 12.4 ^d^	2250.0 ± 15.6 ^b^	N.D. ^g^	2000.0 ± 14.2 ^c^	3000.0 ± 17.6 ^a^	78.0 ± 0.68 ^e^	50.0 ± 0.48 ^f^

* N.D. = Not detected. The values are the average of triplicate samples (n = 3) ± S.D. (*p* ≤ 0.05). The superscript letters represent the significant differences as analyzed by ANOVA; ^†^Ciprofloxacin and fungone were used as reference standards for bacterial and fungal strains, respectively.

## Supplementary Materials

Supplementary materials can be accessed at: http://www.mdpi.com/1420-3049/17/12/14275/s1.
