# Supplementary material for: In Vitro Antimicrobial, Antioxidant, Cytotoxicity and GC-MS Analysis of Mazus goodenifolius
Source: Molecules. 2012 Dec 3;17(12):14275–8. doi: 10.3390/molecules171214275 (PMC6268932; doi:10.3390/molecules171214275)
Supplement: Supplementary file 1 [file molecules-17-14275-s001.docx]

**Supplementary Data**

**Figure 1.** GC*-MS* chromatogram *M. Goodenifolius* essential oil.


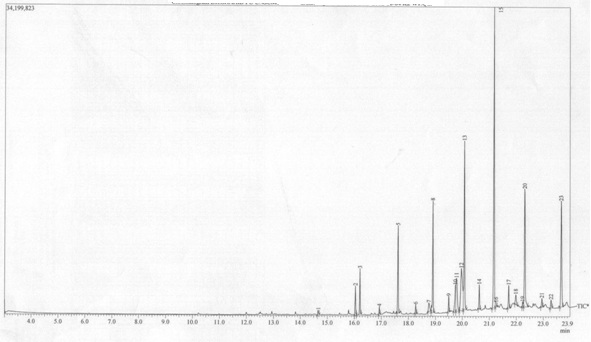


**Figure 2.** Structures of some major compounds identified by GC-MS analysis in  *M. Goodenifolius* Essential oil
